# Application of the patient-reported outcomes continual reassessment method to a phase I study of radiotherapy in endometrial cancer

**DOI:** 10.1515/ijb-2022-0023

**Published:** 2022-11-17

**Authors:** Nolan A. Wages, Bailey Nelson, Jordan Kharofa, Teresa Meier

**Affiliations:** Department of Biostatistics, Virginia Commonwealth University, Richmond, VA, USA; Massey Cancer Center, Virginia Commonwealth University, Richmond, VA, USA; Department of Radiation Oncology, University of Cincinnati, Cincinnati, OH, USA

**Keywords:** continual reassessment method, dose-finding, patient-reported outcomes

## Abstract

This article considers the concept of designing Phase I clinical trials using both clinician- and patient-reported outcomes to adaptively allocate study participants to tolerable doses and determine the maximum tolerated dose (MTD) at the study conclusion. We describe an application of a Bayesian form of the patient-reported outcomes continual reassessment method (PRO-CRMB) in an ongoing Phase I study of adjuvant hypofractionated whole pelvis radiation therapy (WPRT) in endometrial cancer (NCT04458402). The study’s primary objective is to determine the MTD per fraction of WPRT, defined by acceptable clinician- and patient-reported DLT rates. We conduct simulation studies of the operating characteristics of the design and compared them to a rule-based approach. We illustrate that the PRO-CRMB makes appropriate dose assignments during the study to give investigators and reviewers an idea of how the method behaves. In simulation studies, the PRO-CRMB demonstrates superior performance to a 5 + 2 stepwise design in terms of recommending target treatment courses and allocating patients to these courses. The design is accompanied by an easy-to-use R shiny web application to simulate operating characteristics at the design stage and sequentially update dose assignments throughout the trial’s conduct.

## 1 Introduction

This article considers the concept of designing Phase I clinical trials using both clinician and patient-reported outcomes (PROs) to adaptively allocate study participants to tolerable doses and determine the maximum tolerated dose (MTD) at study conclusion. In addition to the National Cancer Institute Common Terminology Criteria for Adverse Events (NCI-CTCAE) for capturing dose-limiting toxicities (DLTs) evaluated by clinicians, the patient-reported outcomes (PRO)-CTCAE provides an instrument for capturing PRO-DLTs that can also be used in determining the MTD. The PRO-CTCAE is made up of 124 items, including 78 symptomatic toxicities from the CTCAE [1]. Whereas many CTCAE categories, such as laboratory values, cannot be scored by the patients, the PRO-CTCAE (scored from 1-5) provides complementary data to the NCI-CTACE in evaluating overall tolerability. For example, grade 3 nausea on NCI-CTCAE is “inadequate oral caloric or fluid intake; tube feeding; TPN or hospitalization indicated”, while a score of 3 on the PRO-CTCAE scale for nausea is “moderate” severity. Patients with NCI-CTCAE grade 3 nausea will likely have a 4 or 5 score on PRO-CTCAE (severe or very severe). NCI-CTCAE looks at toxicity from the clinician’s point of view with more objective data, and the PRO-CTCAE offers subjective information from the patient’s point of view. As another example, NCI-CTCAE grading for diarrhea is based on the number of stools per day above a patient’s baseline (quantitative data), while patients use a qualitative scale on PRO-CTCAE to report events. So, a patient may score 4 on the PRO-CTCAE scale for “frequently having diarrhea”, but the NCI-CTCAE grade would be 2 for having 4 stools per day above their baseline. This participant would have a DLT per PRO-CTCAE but not based upon NCI-CTCAE. If a particular dose results in a large percentage of participants experiencing PRO-DLTs, a design should account for these events to accrue future participants to the study on more patient tolerable doses.

Lee, Lu, and Cheng [2] propose three extensions of the likelihood-based, two-stage, continual reassessment method [3] that use a NCI-DLT endpoint and a PRO-DLT endpoint in adaptive dose assignments. Their work, termed the patient-reported outcomes continual reassessment method (PRO-CRM), introduced a marginal modeling approach in which clinician and patient outcomes are modeled separately. The other two methods impose a constraint using a joint outcome defined by clinician and patient outcomes and modeled either jointly or marginally. The joint outcome with joint modeling approach is akin to estimating the MTD using the CRM with multiple toxicity constraints [4], and adds to a class of multiple-type multiple-toxicity phase I dose-finding designs [5–10]. Simulation results in Lee et al. [2] indicate that the marginal approach has good statistical properties and is simple to implement. The joint outcome based on marginal modeling demonstrates better performance but is more challenging to execute.

This article describes an application of a Bayesian form of the PRO-CRM [2], using the marginal modeling framework, to an ongoing Phase I study of adjuvant hypofractionated whole pelvis radiation therapy in endometrial cancer (NCT04458402). This trial, which opened for accrual at the University of Cincinnati Cancer Center in March 2021, is evaluating two hypofractionated WPRT regimens in gynecologic cancer delivered in 10 or 15 fractions. The study’s primary objective is to determine the maximum tolerated dose (MTD) per fraction, defined by acceptable acute clinician-reported gastrointestinal (GI) and genitourinary (GU) toxicity and patient-reported GI toxicity, of WPRT from two biologically equivalent study dose levels: 41.25 Gy in 15 fractions for dose level 1 and 38 Gy in 10 fractions for dose level 2. When condensing equivalent radiation fractionation schemes into less fractions, there has historically been a concern that this may result in more severe side effects so these doses are considered ordered with respect to their respective DLT probabilities. This application of PRO-CRM focuses on the marginal approach because, in addition to being easy to execute, clinical investigators for the study were comfortable specifying a target PRO-DLT rate to define the MTD. The design is accompanied by an R shiny web application at http://uvatrapps.uvadcos.io/pro-crm/.

## 2 Methods

Both clinician- and patient-reported DLT, termed C-DLT and P-DLT, respectively, are binary outcomes. Estimation is based on separate models for the probability of C-DLT and the probability of P-DLT, as well as the accumulated C-DLT and P-DLT data at each dose level, to sequentially allocate each new patient cohort. For general application, consider a Phase I trial evaluating *J* discrete study dose levels *X* = {*x*_1_, …, *x*_*J*_}. We index two binary endpoints by *i* = 1 for a C-DLT and *i* = 2 for a P-DLT, with *Y*_1_ = 1 indicating the occurrence of a C-DLT, *Y*_2_ = 1 indicating a P-DLT, and 0 otherwise. Denote the probability of observing the outcome *Y*_*i*_ = 1 at dose level *x*_*j*_ by *π*_*i*_(*x*_*j*_), *i* = 1, 2; *j* = 1, …, *J*. The primary objective of the study is to determine the MTD, *γ* ∈ {*x*_1_, …, *x*_*J*_}, defined as the minimum dose with a C-DLT rate closest to the clinician-reported target C-DLT rate of *ϕ*_1_ and the dose with a P-DLT rate closest to the patient-reported target P-DLT rate of *ϕ*_2_ so that

$$\gamma =\min\left(x_{1}^{*},x_{2}^{*}\right)$$

where $x_{i}^{*}\in x_{1},\ldots ,x_{J}$ and $x_{i}^{*}=\mathrm{arg min}|\pi_{i}\left(x_{j}\right)-\phi_{i}|$ . The goal, both within and after the study, is to identify *γ*. We model the probability of observing *Y*_*i*_ = 1 at dose level *x*_*j*_ via an empiric working model

(1) $$\pi_{i}\left(x_{j}\right)=\mathrm{Pr}\left(Y_{i}=1|\;x_{j}\right)=F\left(x_{j},\beta_{i}\right)\approx {\left(p_{i}\left(x_{j}\right)\right)}^{\exp\left(\beta_{i}\right)}$$

Where the *p*_*i*_(*x*_*j*_) are a set of pre-specified constants representing initial guesses of the outcome probabilities at each dose level. These constants, termed the skeleton of the model, are calibrated to yield good design operating characteristics. The prior distribution on *β*_*i*_ is *g*(*β*_*i*_). At any point in the study, the data for outcome *i* at dose level *x*_*j*_ are *D*_*i*_ = {(*y*_*ij*_, *n*_*ij*_): *i* = 1, 2; *j* = 1, …, *J*}, where *y*_*ij*_ is the number of observed occurrences of the outcome *Y*_*i*_ at dose level *x*_*j*_, and *n*_*ij*_ is the number of patients evaluated for the occurrence of the outcome *Y*_*i*_ at dose level *x*_*j*_. For outcome *i*, the likelihood function is given by

$$L\left(D_{i}|\beta_{i}\right)\propto \overset{J}{\underset{j=1}{\prod }}{\left(F\left(x_{j},\beta_{i}\right)\right)}^{y_{ij}}{\left(1-F\left(x_{j},\beta_{i}\right)\right)}^{n_{ij}-y_{ij}}.$$

The posterior mean for the probability of outcome *i* at dose level *x*_*j*_ is given by

$${\tilde{\pi }}_{i}\left(x_{j}\right)=\overset{\infty }{\underset{-\infty }{\int }}F\left(x_{j},\beta_{i}\right)\;T\left(\beta_{i}|\;D_{i}\right)\;d\beta_{i},$$

where *T*(*β*_*i*_|*D*_*i*_) is the posterior density of *β*_*i*_ given by

$$T\left(\beta_{i}|\;D_{i}\right)=\frac{L\left(D_{i}|\;\beta_{i}\right)g\left(\beta_{i}\right)}{\overset{\infty }{\underset{-\infty }{\int }}L\left(D_{i}|\;\beta_{i}\right)g\left(\beta_{i}\right)\;d\beta_{i}}.$$

At each accrual decision, we use the estimated probabilities ${\tilde{\pi }}_{i}\left(x_{j}\right)$ for each outcome to guide allocation and identify the MTD after the study. After each cohort inclusion, we estimate $x_{i}^{*}$ using the estimated probabilities ${\tilde{\pi }}_{i}\left(x_{j}\right)$ so that ${\tilde{x}}_{i}^{*}=\mathrm{arg min}|{\tilde{\pi }}_{i}\left(x_{j}\right)-\phi_{i}|$ . We then allocate the next cohort of participants to $\tilde{\gamma }\in \left\{x_{1},\ldots ,x_{J}\right\}$ , where

$$\tilde{\gamma }=\min\left({\tilde{x}}_{1}^{*},{\tilde{x}}_{2}^{*}\right),$$

with the restriction the trial is not allowed to skip dose levels when escalating. Accrual to the study will end after the maximum target sample size of *N* participants has been accrued to the study. The MTD is defined as the dose level $\tilde{\gamma }$ that would have been administered to the next cohort had one been included.

## 3 Phase I trial in endometrial cancer

### 3.1 Design specifications

Acute GI and GU toxicity are being assessed according to both the CTCAE, version 5.0, and the PRO-CTCAE. A C-DLT is defined as an acute grade 3 or higher GI or GU per CTCAE and a P-DLT is defined by GI toxicity with a score of 4 or 5 on the 5-point scale per PRO-CTCAE, occurring within three months of completing WPRT. The MTD is defined as the minimum of the dose with a C-DLT rate closest to the clinician-reported target C-DLT rate of *ϕ*_1_ = 20% and the dose with a P-DLT rate closest to the patient-reported target P-DLT rate of *ϕ*_2_ = 55%. These rates were based on previously published trials of WPRT in the study population [11]. The study is accruing participants in cohorts of size 3. The starting dose level will be dose level 1 (treatment course 41.25 Gy in 15 fractions). The DLT evaluation window is 3 months after completing WPRT. The skeleton values were chosen according to the algorithm of Lee and Cheung [12], using recommended specifications that yield a working model that results in well-performing operating characteristics in a broad range of scenarios. The algorithm is available as a function, **getprior**, within the **R** package **dfcrm** and requires a “spacing” measure *δ* to generate adequate spacing between adjacent values. For a broad range of target DLT rates and number of dose levels studied by Lee and Cheung [12] and Cheung [13], the optimal range of *δ* is typically between 0.04 and 0.10. For this study, we used a value of *δ* = 0.05 to generate the skeletons for each outcome. The resulting skeleton values are *p*_1_(*x*_*j*_) = (0.20, 0.31) for C-DLT probabilities and *p*_2_(*x*_*j*_) = (0.55, 0.64) for P-DLT probabilities. For both outcomes, the prior distribution *g*(*β*_*i*_) on the parameter *β*_*i*_ is specified by a zero-mean Normal distribution, which is common to CRM designs that employ a class of empiric models such as (1). According to Cheung [13], there are two practical advantages for choosing a normal distribution in this setting. The first is that posterior computations using Gauss–Hermite quadrature [14] under the parametrization (1) are accurate, and the second is that the Bayesian CRM utilizing a class of one-parameter models that includes (1) is invariant to the mean of a prior that forms a location-scale family. This property allows for the setting of the prior mean to be zero and the prior to be completely specified by its standard deviation, which simplifies the calibration process. For each outcome, we selected a zero-mean Normal distributions using the algorithm of Lee and Cheung [15] for computing the least informative prior variance. The resulting prior distributions were *β*_1_ ∼ *N*(0, 1.60) and *β*_2_ ∼ *N*(0, 1.58).

### 3.2 Sample size and accrual

Accrual to the study will be halted and trigger a review by the study investigators and the data safety monitoring committee (DSMC) to determine if the study should be modified or permanently closed to further accrual according to the stopping rules in Table 1. For each DLT-type (NCI and PRO), the study will be stopped for safety if the observed DLT rate at the lowest study dose level is greater than or equal to the number of DLTs out of the following number of patients treated at the lowest study dose level (Table 1). These stopping guidelines are based on whether the lower limit of an Agresti and Coull [16] binomial confidence interval (with 70% confidence) for the lowest study dose level exceeds the target rate *ϕ*_*i*_ for each outcome. The following bounds were generated using the web application at https://uvatrapps.shinyapps.io/pro-crm/.

**Table 1:** Stopping guidelines for excessive toxicity at the lowest dose level based on a 70% Agrest-Coull [16] binomial confidence interval.

	Number of participants	Boundary (i.e., number of DLTs)
C-DLT	3–5	≥2
	6–8	≥3
	9–12	≥4
	13–15	≥5
P-DLT	3	≥3
	4–5	≥4
	6	≥5
	7–8	≥6
	9	≥7
	10–11	≥8
	12	≥9
	13–14	≥10
	15	≥11

The MTD is defined as the study dose level $\tilde{\gamma }\in \left\{x_{1},x_{2}\right\}$ that is recommended after the maximum target sample size of *N* = 15 participants is accrued to the study. All patients who are enrolled in the study and receive their assigned dose are considered evaluable for toxicity. Additional participants will be enrolled to replace any participants who are enrolled but do not receive treatment. Accrual is estimated at one participant every other month; thus, accrual to the study should be completed in 24–30 months. After adjusting for an approximate 5% drop-out/ineligibility rate, the maximum target accrual should not exceed 16 participants.

## 4 Results

### 4.1 Illustration

**Figure 1:**
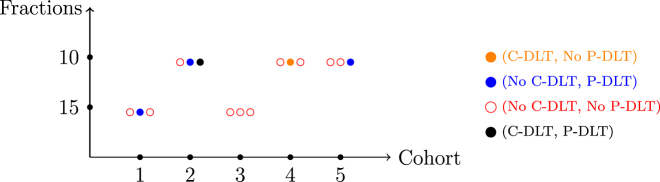
A simulated sequential trial illustrating the described dose-finding design.

In this section, we illustrate the behavior of the design described under a set of true C-DLT and P-DLT probabilities, which will serve as Scenario 1 in our simulation studies in the following section. The target C-DLT rate is *ϕ*_1_ = 20% and the target P-DLT rate is *ϕ*_2_ = 55%. The set of true C-DLT probabilities are {0.05, 0.15} and the set of the true P-DLT probabilities are {0.18, 0.35}, indicating that both dose levels are safe. The data from the entire simulated trial are provided in Figure 1. The first three eligible participants are administered dose level 1 (15 fractions), with 0 C-DLTs and 1 P-DLT observed. Based on this data, the model-based C-DLT probability estimates are {0.01, 0.02} and the P-DLT probability estimates are {0.43, 0.55}. These estimates indicate that dose level 2 has estimated DLT rates closest to the respective target rates for each endpoint, so the trial escalates to dose level 2 [17]. One participant in the second cohort experiences a C-DLT and P-DLT, and another experiences a C-DLT only. The resulting model-based C-DLT and P-DLT estimate are {0.13, 0.22} and {0.497, 0.604}, respectively, indicating that the trial should return to dose level 1 after observing these DLTs. The third cohort receives dose level 1 and no DLTs of either type are observed, and the trial quickly returns to dose level 2. In the fourth cohort, one C-DLT and no P-DLTs are observed. Based on this data, the model-based C-DLT probability estimates are {0.12, 0.21} and the P-DLT probability estimates are {0.22, 0.34}. These estimates indicate that dose level 2 has estimated DLT rates closest to the respective target rates for each endpoint, so the trial remains at dose level 2. The trial remains at dose level 2 for the fifth and final cohort and one additional P-DLT is observed in the last three participants. The final model-based C-DLT and P-DLT estimate are {0.09, 0.17} and {0.22, 0.34}, respectively. Overall, in this simulated trial (Figure 1), *N* = 15 patients were treated, yielding a final MTD recommendation of dose level 2 (10 fractions).

### 4.2 Operating characteristics

Simulations were run to display the performance of the design characteristics. In the initial meeting with the statistical team about the Phase I endometrial cancer trial, the clinical team presented the 5 + 2 design [18] as a possible approach to the problem since they had seen it recently implemented in a published clinical trial (RTOG 0117 [19]). However, they were open to hearing about other design options from the statistical team. At that time in early 2020, the only existing method that formally incorporated PROs was the PRO-CRM [2] approach, which first appeared online in December 2019. So when the statistical team suggested a Bayesian PRO-CRM approach for the trial, we felt it useful to compare it to the initially suggested 5 + 2 design in order to show which is the better design choice. With the 5 + 2 design, the study would begin by enrolling five patients in Cohort 1. Transition to Cohort 2 will be based on the following rules based on NCI-CTCAE and PRO-CTCAE Events:

– If 0 of the 5 patients have a DLT per CTCAE and 2 or less of the 5 patients have a DLT per PRO-CTCAE, then accrual into Cohort 2 will begin.
– If 1 of the 5 patients in Cohort 1 has a DLT per CTCAE and/or 3 of the 5 patients have a DLT per PRO-CTCAE, 2 more patients will be enrolled in Cohort 1 for a total of 7 patients.
  – If 1 of the 7 patients has a DLT per CTCAE and 3 or fewer patients have a DLT per PRO-CTCAE, then accrual into Cohort 2 will begin.
  – The accrual goal for cohort 2 is 7 patients (total *n* = 12–14). If 2 or more of the 7 patients have a DLT per CTCAE or 4 or more of the 7 patients have a DLT per PRO-CTCAE, Cohort 1 will be considered the MTD.
– If 2 or more patients have a DLT per CTCAE and/or 4 or more patients have a DLT per PRO-CTCAE in cohort 1, then the study will end, and we will conclude that 15 fraction RT (cohort 1) is not safe or feasible.

We assumed a set of true DLT probabilities for each endpoint (C-DLT and P-DLT). We evaluated each design under a broad range of hypothesized probability scenarios. Under each scenario, we

1. Simulated trial data for the PRO-CRMB and 5 + 2 stepwise designs.
2. Recorded which dose level (fractions course) was chosen as MTD.
3. Repeated trial simulation many times (i.e., 10,000).
4. Tabulated the percentage of times each course is chosen as the MTD over the 10,000 simulated trials and the percentage of times the trial stopped early for safety. The maximum sample size is *n* = 15 for PRO-CRMB and *n* = 14 for 5 + 2 design.
5. Recorded the average number of participants treated on each course and the average sample size over 10,000 simulated trials

The confidence level used to define the safety stopping guidelines for the PRO-CRMB design was 70%. Tables 2 and 3 report the true C-DLT probability at each dose level, the true P-DLT probability at each dose level, the percentage of trials in which each dose level was recommended as the MTD, the average number of participants treated at each dose level, the average sample size, and the percentage of trials stopped early for safety.

**Table 2:** Percentage of MTD selection at each course for the 5 + 2 design and the Bayesian PRO-CRM with cohorts of size 3.

Scenario		15 fractions	10 fractions	% stopped for safety
1	True C-DLT probability	0.05	**0.15**	
	True P-DLT probability	0.18	**0.35**	
	PRO-CRMB	13.0	**85.5**	1.6
	5 + 2 stepwise	40.8	**53.0**	6.2
2	True C-DLT probability	**0.20**	0.40	
	True P-DLT probability	**0.18**	0.35	
	PRO-CRMB	**55.4**	19.1	25.4
	5 + 2 stepwise	**50.2**	7.6	42.2
3	True C-DLT probability	0.10	**0.20**	
	True P-DLT probability	0.35	**0.55**	
	PRO-CRMB	36.0	**53.2**	10.8
	5 + 2 stepwise	54.2	**16.0**	29.8
4	True C-DLT probability	**0.08**	0.15	
	True P-DLT probability	**0.50**	0.65	
	PRO-CRMB	**44.8**	31.5	23.7
	5 + 2 stepwise	**43.2**	7.5	49.3
5	True C-DLT probability	0.08	0.15	
	True P-DLT probability	0.65	0.75	
	PRO-CRMB	37.7	7.1	**55.2**
	5 + 2 stepwise	22.1	1.2	**76.7**
6	True C-DLT probability	0.40	0.45	
	True P-DLT probability	0.25	0.35	
	PRO-CRMB	17.5	2.9	**79.6**
	5 + 2 stepwise	21.6	1.9	**76.6**

Bold font indicates the level that is the MTD corresponding to a target C-DLT rate of 20% and a target P-DLT rate of 55%.

**Table 3:** Average number of patients treated at each course for the 5 + 2 design and the Bayesian PRO-CRM with cohorts of size 3.

Scenario		15 fractions	10 fractions	Average trial size
1	True C-DLT probability	0.05	**0.15**	
	True P-DLT probability	0.18	**0.35**	
	PRO-CRMB	5.6	**9.2**	14.8
	5 + 2 stepwise	5.5	**6.6**	12.1
2	True C-DLT probability	**0.20**	0.40	
	True P-DLT probability	**0.18**	0.35	
	PRO-CRMB	**9.4**	3.7	13.1
	5 + 2 stepwise	**5.9**	4.0	9.9
3	True C-DLT probability	0.10	**0.20**	
	True P-DLT probability	0.35	**0.55**	
	PRO-CRMB	8.4	**5.5**	13.9
	5 + 2 stepwise	5.9	**4.9**	10.8
4	True C-DLT probability	**0.08**	0.15	
	True P-DLT probability	**0.50**	0.65	
	PRO-CRMB	**9.2**	3.6	12.8
	5 + 2 stepwise	**6.0**	3.5	9.5
5	True C-DLT probability	0.08	0.15	
	True P-DLT probability	0.65	0.75	
	PRO-CRMB	8.5	1.4	9.9
	5 + 2 stepwise	6.0	1.6	7.6
6	True C-DLT probability	0.40	0.45	
	True P-DLT probability	0.25	0.35	
	PRO-CRMB	7.3	1.1	8.4
	5 + 2 stepwise	5.7	1.6	7.3

Bold font indicates the level that is the MTD corresponding to a target C-DLT rate of 20% and a target P-DLT rate of 55%.

In Scenario 1, both treatment courses have DLT probability rates below the respective safety thresholds <20% and <55% for each endpoint. The assumed MTD is 10 fractions with an NCI-CTCAE DLT rate of 15% and PRO-CTCAE DLT rate of 35%. The PRO-CRMB has a 32.5% higher chance of selecting 10 fractions as the MTD in this scenario compared to the 5 + 2 design (85.5% vs. 53%). On average, the PRO-CRMB design treats more patients at the hypothesized MTD than the 5 + 2 design (9.2 vs. 6.6). The average sample size is 12.1 patients for the 5 + 2 design and 14.8 for the PRO-CRMB design. In Scenario 2, 10 fractions has an NCI-CTCAE DLT rate >20% , while both courses have PRO-CTCAE DLT rates ≤35% . The assumed MTD is 15 fractions with an NCI-CTCAE DLT rate of 20% and a PRO-CTCAE DLT rate of 18%. The PRO-CRMB design has a 5.2% higher chance of selecting 15 fractions as the MTD in this scenario compared to the 5 + 2 design (55.4% vs. 50.2%). Although a safe dose level (15 fractions) exists in this scenario, the 5 + 2 design incorrectly stops early for safety in 42.2% of the simulated trials. On average, the PRO-CRMB design treats more patients at the hypothesized MTD than the 5 + 2 design (9.4 vs. 5.9). The average sample size is 9.9 patients for the 5 + 2 design and 13.1 for the PRO-CRMB design. In Scenario 3, 10 fractions has DLT rates equal to the respective thresholds for each endpoint. The assumed MTD is 10 fractions with an NCI-CTCAE DLT rate of 20% and PRO-CTCAE DLT rate of 55%. The PRO-CRMB design has a 37.2% higher chance of selecting 10 fractions as the MTD in this scenario compared to the 5 + 2 design (53.2% vs. 16%). On average, the PRO-CRMB design treats more patients at the hypothesized MTD than the 5 + 2 design (5.5 vs. 4.9). The average sample size is 10.8 patients for the 5 + 2 design and 13.9 for the PRO-CRMB design.

In Scenario 4, 10 fractions has a PRO-CTCAE DLT rate >55% , while both courses have NCI-CTCAE DLT rates <20% . The assumed MTD is 15 fractions with an NCI-CTCAE DLT rate of 8% and a PRO-CTCAE DLT rate of 50%. The PRO-CRMB design has a 1.6% higher chance of selecting 15 fractions as the MTD in this scenario compared to the 5 + 2 design (44.8% vs. 43.2%). Although a safe dose level (15 fractions) exists in this scenario, the 5 + 2 design incorrectly stops early for safety in 49.3% of the simulated trials. On average, the PRO-CRMB design treats more patients at the hypothesized MTD than the 5 + 2 design (9.2 vs. 6.0). The average sample size is 9.5 patients for the 5 + 2 design and 12.8 for the PRO-CRMB design. In Scenario 5, both courses have PRO-CTCAE DLT rates >55% . There is no assumed MTD, and the correct decision is to stop the trial early for safety. The 5 + 2 design terminates early in a higher percentage of simulated trials than the PRO-CRMB design (76.7% vs. 55.2%). The average sample size is 7.6 patients for the 5 + 2 design and 9.9 for the PRO-CRMB design. In Scenario 6, both courses have NCI-CTCAE DLT rates >20% . There is no assumed MTD, and the correct decision is to stop the trial early for safety. PRO-CRMB stops early in a slightly higher percentage of the simulated trials than the 5 + 2 design (79.6% vs. 76.6%). The average sample size is 7.3 patients for the 5 + 2 design and 8.4 for the PRO-CRMB design. The stopping rules for PRO-CRMB can be adjusted, if needed, by using a different value for the confidence level. Still, the clinical team was comfortable going forward with the design described based on these simulation results. It is clear from examining the results in Tables 2 and 3 that the proposed Bayesian PRO-CRMB method performs well in terms of recommending target treatment courses and allocating patients to these courses.

## 5 R shiny web application

The web application is written in the R programming language [20] and is made freely available using the Shiny package [21]. Access to the application online is available at https://uvatrapps.shinyapps.io/pro-crm/. The R code for the application can be downloaded by locating the “R code” section at https://nolanwages.faculty.virginia.edu/software. The application has a simple web interface with the capability to:

1. Simulate operating characteristics for the Bayesian form of the PRO-CRM,
2. Compute the recommended dose level for the next patient cohort based on accumulated data,
3. Estimate the maximum tolerated dose (MTD) at the conclusion of the study.

### 5.1 Simulation input

The simulation function generates the operating characteristics of the Bayesian PRO-CRM based upon the user specifying the following set of input parameters.

1. An assumed set of true NCI-CTCAE DLT probabilities, separated by commas (i.e., 0.05, 0.15).
2. An assumed set of true PRO-CTACE DLT probabilities, separated by commas (i.e., 0.18, 0.35).
3. The target NCI-CTACE DLT probability (i.e., 0.20).
4. The target PRO-CTACE DLT probability (i.e., 0.55).
5. The cohort size required before the next model-based update. Cohort size may be 1, 2, or 3 patients (i.e., 3).
6. The maximum sample size for the study. This number should be a multiple of the cohort size entered above (i.e., 15).
7. The number of simulations. A minimum of 1000 is recommended (i.e., 10,000).
8. The index of the starting dose level. Note: The index of lowest dose level is always 1. If the design allows for ‘minus’ dose levels (i.e. −2, −1 dose levels), then the index of the starting dose should account for these lower levels (i.e. if a −1 dose level is allowed, the index of the starting dose is 2).
9. The total number of patients treated on any dose required to stop the trial. At any point in the trial, if the recommendation is to assign the next cohort to a dose that already has the entered number of patients treated on the dose, the study is stopped and the recommended dose is declared the optimal dose. If the entered number is larger than the maximum sample size, each trial will accrue to the maximum sample size (i.e., 16).
10. The confidence level for safety stopping rule at the lowest study dose level (i.e., 0.80).
11. The seed of the random number generator (i.e., 34,895).
12. The variance of the normal prior for the NCI-DLT probability model (i.e., 1.60).
13. The variance of the normal prior for the PRO-DLT probability model (i.e., 1.58).

The simulation results will be generated by clicking the **Run Simulation Study** button. It is also of interest for investigators to be able to conduct a trial with the using the app. That is, given accumulated DLT data for all patients on each dose level, what dose would be recommended for the next entered patient cohort?

### 5.2 Implementation input

For implementation, the app relies upon the user specifying the following set of input parameters:

1. Design/protocol information
  (a) The target NCI-CTACE DLT probability (i.e., 0.20).
  (b) The target PRO-CTACE DLT probability (i.e., 0.55).
  (c) The variance of the normal prior for the NCI-DLT probability model (i.e., 1.60).
  (d) The variance of the normal prior for the PRO-DLT probability model (i.e., 1.58).
2. Observed trial data (do not count “replaced” patients). The length of inputs (a)–(d) should be equal to the number of possible study dose levels
  (a) The number of observed NCI-CTCAE DLTs at each dose level. If none have been observed or a dose level has not yet been tried, enter “0”. (i.e., 0.1).
  (b) The number of patients evaluated for NCI-CTCAE DLT at each dose level. If a dose level has not yet been tried, enter “0”. (i.e., 3.3).
  (c) The number of observed PRO-CTCAE DLTs at each dose level. If none have been observed or a dose level has not yet been tried, enter “0”. (i.e., 0.2).
  (d) The number of patients evaluated for PRO-CTCAE DLT at each dose level. If a dose level has not yet been tried, enter “0”. (i.e., 0.3).
  (e) The most recent dose level administered in the study (i.e., 2).
  (f) The confidence level for safety stopping rule at the lowest study dose level (i.e., 0.80)

The implementation results will be generated by clicking the **Get updated recommended dose level** button.

### 5.3 Simulation output

Of particular interest in simulating operating characteristics is the accuracy of the method under an assumed set of true DLT probabilities and the target DLT rate for each endpoint. Accuracy is typically measured by the percentage of simulated trials in which the true MTD is recommended as the MTD at the conclusion of the study. This is commonly termed the percentage of correct selection (PCS). Also of interest is the safety of the design, which is typically evaluated by how patients are allocated. Safety can be assessed through observing how many patients were allocated, on average, to dose levels at and around the true MTD, as well as by how many patients, on average were treated above the true MTD. Based on the simulation input provided by the user, the application will produce operating characteristics for the Bayesian PRO-CRM using the statistical parameters specified above. The results output:

1. The percentage of trials in which each dose was selected as the MTD.
2. The average number of NCI-CTCAE DLTs observed at each dose level.
3. The average number of PRO-CTCAE DLTs observed at each dose level.
4. The average number of patients treated at each dose level.
5. The percentage of trials stopped for NCI-CTCAE safety.
6. The percentage of trials stopped for PRO-CTCAE safety.
7. The skeleton of the working model used.

**Figure 2:**
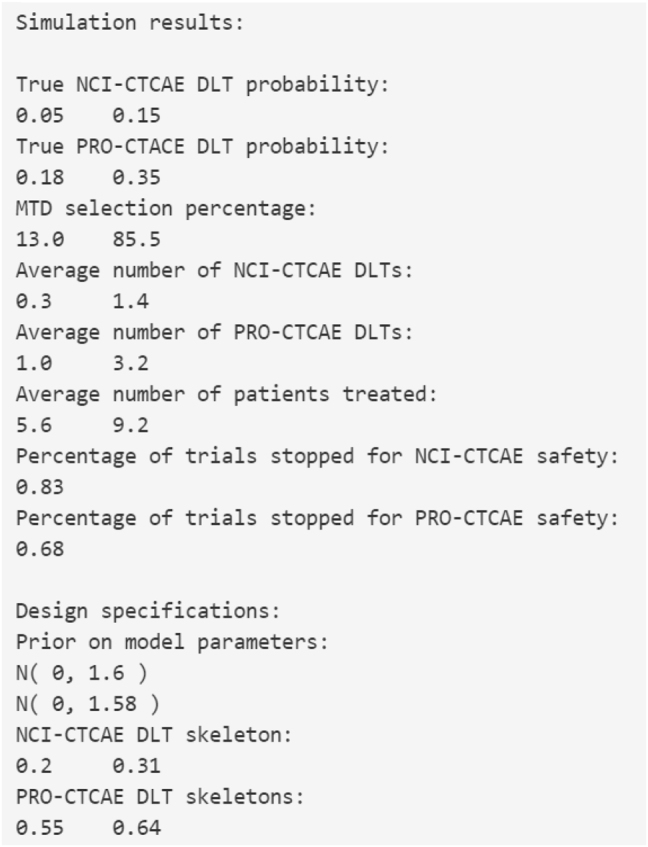
Output for the simulation component of the PRO-CRM web application.

As an example, consider the input specifications in Section 5.1. Based on 1000 simulated trials of 24 patients, the output in Figure 2 is generated. These results can be copied and pasted into a protocol document. The skeleton used in each simulated trial is {0.20, 0.31}. Targeting *ϕ*_1_ = 0.20 and *ϕ*_2_ = 0.55, the assumed MTD under the set of true DLT probabilities is dose level 2, with a true NCI-CTCAE DLT probability of 0.15 and a true PRO-CTCAE DLT probability of 0.35. Dose level 2 is selected as the MTD in 85.5% of simulated trials, while 9.2 of 14.8 patients on average are treated at the true MTD in this scenario. The results in Table 3 can be reproduced exactly by any user by inputting the exact same design specifications in Section 5.1, provided that the same random seed is used to generate the outcomes in each simulated trial.

### 5.4 Implementation output

**Figure 3:**
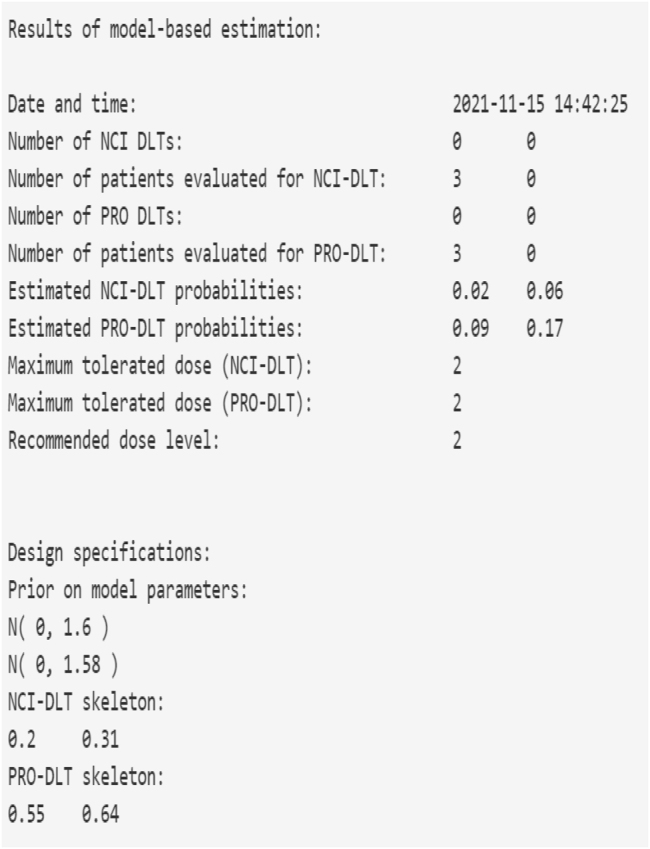
Output for the implementation component of the PRO-CRM web application. After the accrual of each new patient cohort, the model-based DLT probability estimates and recommended dose level for the next accrued cohort are updated.

Figure 3 illustrates the application’s ability to update model-based estimates for the DLT probabilities and the recommended dose for the next entered cohort. Suppose we begin a study at dose level 1, and are accruing to the study in patient cohorts of size 3. The target DLT rates are *ϕ*_1_ = 20% and *ϕ*_2_ = 55%, and the first three entered patient do not experience a DLT. Based on the three non-DLT observations at dose level 1, we can see that the estimated DLT probabilities for each outcomes have been updated, indicating that dose level 2 is closest to the target dose, with an estimated NCI-DLT rate 0.06 and an estimated NCI-DLT rate 0.17. The Recommended dose level in Figure 3 is level 2. The user would then update Current dose level in the input parameters to dose level 2, and observe the DLT outcomes (yes/no) for the second entered patient cohort. The implementation portion of the application can be used to sequentially provide model-based dose recommendations for trial conduct in real studies. It is also useful in providing tables of the early design behavior (i.e., dose transition pathways) in the protocol statistical section, so that reviewers get an idea of how the design allocates early in the study. The date and time each implementation output was generated is given at the top of the output, so that each recommendation can be properly documented.

### 5.5 Safety stopping bounds

The stopping bound component generates NCI-DLT and PRO-DLT stopping bounds at the lowest study dose level based on Agresti–Coull binomial confidence interval estimation, based on the following input parameters.

1. The target NCI-CTACE (or PRO-CTCAE) DLT probability (i.e., 0.20 or 0.55).
2. The maximum sample size for the study (i.e., 15).
3. The confidence level for safety stopping rule at the lowest study dose level (i.e., 0.70).

These input parameters would generate the stopping bounds reported in Table 1 for each outcome.

## 6 Conclusions

The accessibility of more rigorous mechanisms for capturing DLT data directly from patients has led to an increasing interest in integrating PROs into early-phase trials to better define treatment tolerability [22–24]. Several publications indicate a high rate of disagreement between clinician- and patient-reported symptoms, with patients reporting symptoms more frequently and with more severity [25–29]. The PRO-CTCAE events are more sensitive to the patient experience relative to the NCI-CTCAE events. Failing to capture and utilize these data in early development can lead to treatment regimens that may not be tolerable for patients being carried forward into middle and late development based only on clinician assessment. It is beneficial for a method to have the flexibility of allocating patients to lower, more tolerable regimens if a large number of PRO-DLT events are seen at higher doses. We must have patient-reported upper bound criteria in evaluating new therapies early. If there are concerning signals for low tolerance to treatment from a patient’s perspective, it would be valuable to know early on before the therapy advances further.

The growth of novel methods in early-phase dose-finding has been rapid in the last decade, yet execution of innovative designs remain uncommon [30, 31]. The use of more innovative approaches is being encouraged by the FDA and others [32, 33]. This article has described the application of a novel adaptive strategy that accounts for clinician- and patient-reported DLTs to an ongoing Phase I trial of adjuvant hypofractionated WPRT in endometrial cancer (NCT04458402). Simulation studies were performed to justify and evaluate the performance of the design characteristics. The simulation results in Tables 2 and 3 demonstrate the method’s ability to effectively recommend desirable treatment courses, defined by acceptable toxicity according to the NCI-CTCAE and PRO-CTCAE, in a high percentage of trials with manageable sample sizes.

Software in the form of an R Shiny web application for both simulation of design operating characteristics and direct protocol implementation of the method is available at https://uvatrapps.shinyapps.io/pro-crm/. The method we applied in this work can be viewed as an extension of the CRM, utilizing working models to account for NCI-DLT and PRO-DLT probabilities in assigning participants to doses and identifying the MTD. This ability increases the flexibility of CRM designs, enabling them to handle more complex dose-finding problems. For future work, it would be possible to extend existing CRM adaptations for contemporary dose-finding problems to incorporate PROs into the modeling and dose assignment algorithm. For instance, the weighted likelihood approach employed by the time-to-event CRM (TITE-CRM [34]) could be developed for a PRO-DLT endpoint. The specification of multiple working models in order to accommodate partially ordered drug combination surfaces (POCRM [35]) could be extended to the PRO toxicity setting. In the Phase I/II framework (Wages and Tait [36]), PRO-DLTs could serve as an additional endpoint to traditional toxicity and efficacy endpoints in guiding adaptive dose assignments. The numerical results presented in the simulation studies, such as the distribution of sample size and frequency of early trial termination, are the type of simulation information that improves understanding, acceptance, and approval of novel designs such as the one described in this manuscript [37, 38]. This support for adaptive methods will augment efficient early-phase trial design in contemporary dose-finding studies. Well-performing dose-finding designs can have a tremendous impact on the future of oncology care [39].
